# ﻿*Phenasurya daeng*, a new genus and species of plexippine jumping spider (Salticidae, Plexippini, Plexippina) from Thailand

**DOI:** 10.3897/zookeys.1259.169914

**Published:** 2025-11-12

**Authors:** Kiran Marathe, Łukasz Trębicki, Katarzyna Janik-Superson, Abdulloh Samoh, Junxia Zhang, Wayne P. Maddison

**Affiliations:** 1 Department of Zoology and Beaty Biodiversity Museum, University of British Columbia, 6270 University Boulevard, Vancouver, British Columbia, V6T 1Z4, Canada; 2 Department of Invertebrate Zoology and Hydrobiology, Faculty of Biology and Environmental Protection, University of Lodz, Lodz, Poland, Banacha 12/16, 90-237, Lodz, Poland; 3 Centre for Digital Biology and Biomedical Science–Biobank Lodz, Faculty of Biology and Environmental Protection, University of Lodz, Lodz, Poland; 4 Princess Maha Chakri Sirindhorn Natural History Museum (PSUNHM), Prince of Songkla University, Kho Hong, Hat Yai, Songkhla, 90110, Thailand; 5 Key Laboratory of Zoological Systematics and Application, College of Life Sciences and Hebei Basic Science Center for Biotic Interaction, Hebei University, Baoding, Hebei 071002, China; 6 Hebei Basic Science Center for Biotic Interaction, Hebei University, Baoding, Hebei 071002, China; 7 Department of Botany, University of British Columbia, 6270 University Boulevard, Vancouver, British Columbia, V6T 1Z4, Canada

**Keywords:** Araneae, biodiversity, Indochina, phylogenomics, systematics, taxonomy, UCE

## Abstract

We describe a new plexippine genus and new species, *Phenasurya daeng***gen. et sp. nov.**, with a striking red face, from Thailand. Its placement within Plexippina is supported by ultraconserved element (UCE)-based phylogenomic analyses, supplemented with a broader four-gene analysis. Morphological evidence further justifies its generic status. *Phenasurya daeng* is recovered near cf. *Colopsus* and *Pancorius* but is morphologically distinct from both. These findings reveal a new red-faced lineage and settle the identity of a perplexing jumping spider.

## ﻿Introduction

The jumping spider fauna of Thailand remains incompletely known, with only 50 species in 34 genera formally recorded to date (Seyfulina et al. 2020), a figure that is almost certainly an underestimate. The focal spider of this study—characterized by simple, elegant white markings on a black carapace and abdomen, and a distinctive red face—is among the most photographed and widely distributed jumping spiders in Thailand (e.g., Sim 2024; Babineau 2025; Reinthong 2025), and yet its identification to both genus and tribe has remained unclear (e.g., Sim 2024; Reinthong 2025). Its somewhat rounded appearance and general body markings may recall the salticine *Carrhotus* Thorell, 1891, for example *C. sannio* (Thorell, 1877), whereas its high carapace, robust long legs, and distinctive carapace pattern suggest affinities with common plexippines in the region (e.g., *Evarcha* Simon, 1902, *Burmattus* Prószyński, 1992, and *Pancorius* Simon, 1902).

Material collected during Łukasz Trębicki’s 2020 expedition to Thailand has allowed us to study this red-faced jumping spider and to place it phylogenetically. Our analyses indicate that it is closely related to cf. *Colopsus* and *Pancorius* but morphologically distinct, thereby warranting the establishment of a new genus.

## ﻿Material and methods

### ﻿Materials examined

Specimens were collected during an expedition to Kanchanaburi Province, Thailand, in 2020 (23 January–12 February). Fieldwork was conducted under extremely dry conditions, with persistently high temperatures and multiple wildfires in the region. Collecting was performed using an entomological umbrella; specimens were transferred to glass vials, selected individuals were photographed alive, and all material was subsequently preserved in 96% ethanol.

Specimen collection was conducted under permits issued by the Princess Maha Chakri Sirindhorn Natural History Museum (PSUNHM), Prince of Songkla University, Hat Yai, Thailand, where the type material is deposited.

### ﻿Morphology

A drawing tube attached to a Nikon ME600L compound microscope was used to prepare illustrations. Clove oil was used for clearing the epigyne after digesting the internal epigynal soft tissues with pancreatin (Álvarez-Padilla and Hormiga 2007). Preserved specimens were photographed using a ZEISS Stemi 508 Stereo Microscope attached with ZEISS Axiocam 105 (for bodies). Photographs were stacked using Helicon Focus 8.2.1 Pro. Live specimens were photographed during the fieldwork with a Nikon 1 J5 digital camera (Nikon Corporation, Tokyo, Japan) fitted with the dedicated FT1 adapter and a Nikon AF-S DX Micro NIKKOR 40 mm f/2.8G lens (Nikon Corporation, Tokyo, Japan), using an external flash unit equipped with a diffuser.

Descriptions are based on ethanol-preserved specimens. The descriptions were written with primary reference to the focal specimen indicated, but they apply as far as known to the other specimens examined. Carapace length was measured from the anterior base of the median eyes to the posterior margin of the carapace. The abdomen was measured from its anterior edge to the posterior end of the anal tubercle. All the measurements are in millimetres. Abbreviations used here are as follows: **ALE**, anterior lateral eye; **AME**, anterior median eye; **ECP**, epigynal coupling pocket (hood); **PME**, posterior median eye; **PLE**, posterior lateral eye; **RTA**, retrolateral tibial apophysis.

### ﻿Taxon sampling for UCE phylogenomics

In order to place them phylogenetically, molecular data was obtained for a male and female of *Phenasurya daeng*. These were appended to Marathe et al.’s (2024c)UCE phylogenomic dataset. This plexippine-biased dataset was chosen because Plexippina was the most likely home for *Phenasurya daeng*, which has traits (high carapace, robust legs, and distinctive tegular lobe) consistent with the plexippines. Four other taxa were added, the salticine *Salticus scenicus* (Clerck, 1757) from Zhang et al. (2024), and three plexippines *Gratianna yunnanensis* (China), G. cf. yunnanensis (Thailand; co-collected with *Phenasurya*), and *Yaginumaella medvedevi* Prószyński, 1979. *Salticus* and *Carrhotus* (already in the Marathe et al. 2024c dataset) were included in order to have salticines better represented. This was warranted given the initial assessment of *Phenasurya* as a possible salticine. Additional plexippines are added to resolve *Phenasurya* better within Plexippina. Thus, the final dataset comprised 31 taxa, including 25 plexippines (23 species), two salticines, three harmochirines, and one chrysilline (an outgroup). The details of previously unpublished data from four taxa are provided in Table 1, while information on the other taxa used in the phylogenomic analysis follows Marathe et al. (2024c).

**Table 1.** Specimens used in UCE phylogenomic analysis. Only specimens newly included in this study are listed. Most other specimens were also used in Marathe et al. (2024c), which provides the broader sampling.

Species	Voucher	Sex	Locality	Lat, long
*Gratianna yunnanensis*	JXZ822	♂	China	22.0169, 100.8526
Gratianna cf. yunnanensis	SAL_THAI_0248	♀	Thailand	14.145, 99.319
*Phenasurya daeng*	SAL_THAI_0004	♂	Thailand	14.145, 99.319
*Phenasurya daeng*	SAL_THAI_0251	♀	Thailand	14.145, 99.319
*Yaginumaella medvedevi*	MRB064	♂	China	26.3333, 108.3333

### ﻿Taxon sampling for the four-gene phylogenetic analyses

Given the superficial resemblance of *Phenasurya* to *Carrhotus*, we broadened our salticine sampling to provide the new genus the opportunity to cluster among salticines, particularly as the UCE phylogeny lacks dense salticine representation. Accordingly, we incorporated publicly available salticine data to test the phylogenetic affinities of *Phenasurya* within a more densely sampled salticine phylogeny.

We appended bycatch data for four gene regions, recovered from sequence-capture genomic assemblies in the UCE dataset, to a four-gene matrix with dense salticine sampling. Bycatch retrieval followed a protocol similar to Maddison et al. (2020), whereby local BLAST databases were constructed from SPAdes assemblies (Nurk et al. 2013) for each taxon in the UCE dataset. These assemblies were queried using publicly available COI, 28S, 18S, and H3 sequences from seven salticid species: Aelurillus cf. ater (Kroneberg, 1875), *Bianor maculatus* (Keyserling, 1883), *Colopsus cancellatus* Simon, 1902, *Colopsus ferruginus* Kanesharatnam & Benjamin, 2021, *Hyllus treleaveni* Peckham & Peckham, 1902, *Pancorius athukoralai* Kanesharatnam & Benjamin, 2021, and *Salticus scenicus* (Clerck, 1757). Newly included taxa and their accession numbers are given in the Suppl. materials 1, 2; the remaining taxa follow Marathe et al. (2024c).

### ﻿UCE data

Molecular data was gathered for UCE loci using target enrichment sequencing methods (Faircloth 2017) using the RTA_v3 probeset (Zhang et al. 2023) and following the protocols of Marathe et al. 2024a and Zhang et al. 2024. Raw demultiplexed reads were processed with PHYLUCE v. 1.7.3 (Faircloth 2016), quality control and adapter removal were performed with Illumiprocessor wrapper (Faircloth 2013), and assemblies were created with SPAdes v. 3.14.1 (Nurk et al. 2013) using options at default settings. The UCE loci were recovered using RTA_v3 probeset (Zhang et al. 2023). The downstream processing of recovered loci was performed within the Mesquite v. 4.01 (Maddison and Maddison 2025b).

The recovered UCE loci were compiled, and 50 percent occupancy was applied where loci represented in 16 or less taxa were deleted at this stage. The remaining loci were re-aligned with MAFFT using L-INS-i option (Katoh and Standley 2013). The re-aligned UCE loci were then trimmed with PhyIN (Maddison 2024) with default settings (d = 2, e = true, b = 10, p = 0.5) and a 50% site occupancy filter (Maddison 2024). Final alignment trimming was done using Spruceup (Borowiec 2019) to remove outlier sequences from concatenated MSA.

The concatenated MSA was subsequently deconcatenated. From the deconcatenated individual loci, taxa with very short sequences (threshold 50) were removed along each site/ columns with gaps only characters. From the remaining set, loci ≤ 150 bp in length were also removed. RAxML v. 8.2.12 (Stamatakis 2014) gene trees were inferred under GTRGAMMA model for testing loci suspected to include paralogies as in the analysis of Maddison et al. (2020) based on branch lengths. Loci in which the ratio of the longest branches was ≥ 5 were excluded from the dataset. The final set of loci was concatenated for subsequent phylogenetic analyses.

### ﻿DNA barcoding

Genomic DNA was isolated from 21 specimens (14 *Phenasurya daeng* and 6 Gratianna cf. yunnanensis) included in the four-gene phylogeny using the Chelex method (Casquet et al. 2012). A COI gene fragment was amplified with the primer pair LCOjj/HCOjj, producing five overlapping fragments as described in Querner et al. (2022), and three additional fragments covering the 3′ region (Zhang and Maddison 2013). Purified PCR products were outsourced for sequencing to Macrogen Europe. Sequences were aligned in Geneious v. 11.1.5 (Biomatters Ltd) using MUSCLE (Edgar 2004) with default settings. Genetic distances were estimated under the Kimura two-parameter model (K2P) (Kimura 1980). Phylogenetic support was assessed by bootstrap analysis with 500 replicates (Felsenstein 1985), as implemented in MEGA X v. 12 (Kumar et al. 2024).

### ﻿Phylogenomic analysis

Maximum-likelihood phylogenetic and bootstrap analyses were performed with RAxML using the Zephyr v. 4.01 package (Maddison and Maddison 2025a) in Mesquite on the concatenated, unpartitioned UCE dataset. For the phylogenetic tree inference, GTRGAMMAI model of evolution was used for 10 search replicates. For the bootstrap analysis, RAxML search was used for the 1000 search replicates.

### ﻿Four-gene phylogenetic analysis

The loci were aligned using MAFFT with the L-INS-i option, partitioned by locus, and codon positions were assigned to minimize stop codons for H3 and COI. The sequences were then concatenated in Mesquite. Maximum-likelihood phylogenetic analysis and standard bootstrap analyses—constrained by the UCE topology using the -g option—were performed with IQ-TREE v. 3.0.1 (Wong et al. 2025) using -m MFP+MERGE option on the partitioned concatenated dataset, using the Zephyr package in Mesquite. The rationale for the constrained analysis is that the relationships among the majority of taxa included here are consistent with those in the UCE analysis (with four-gene data for these obtained through bycatch), which are supported by robust data and strong bootstrap values. Therefore, the UCE-derived relationships are considered more reliable than those inferred from an unconstrained four-gene analysis. However, additional salticines and plexippines were allowed to be placed freely within the tree.

### ﻿Data availability

The newly obtained raw sequence reads obtained from UCE capture are stored within the Sequence Read Archive (BioProject: PRJNA1321978), and their accession numbers are listed in the Suppl. materials 1, 2. Concatenated UCE matrix used for phylogenetic and bootstrap analysis, along with trees, are available on the Borealis data repository (https://doi.org/10.5683/SP3/8NWTTI). COI sequences have been deposited in BOLD dataset (https://doi.org/10.5883/DS-SALTHGN1), along with collection data and voucher dorsal picture of each specimen. Unique Barcode Index Numbers (Ratnasingham and Hebert 2013) in the BOLD system (Ratnasingham et al. 2024).

## ﻿Results

### ﻿Phylogenetic results

A total of 3,452 UCE loci were initially recovered. After removing loci represented in 50% or fewer taxa and very short sequences, 3,079 loci remained. Following the removal of matrices ≤ 150 bp in length, 3,031 loci were retained. Of these, 3,013 loci remained after excluding those suspected of containing paralogues based on branch length criteria. These were concatenated into the final matrix, with an aligned length of 1,678,691 base pairs. Each taxon contributed on average ~1.4 million base pairs of sequence data (range: 755,964–1,533,638 bp). Suppl. materials 1, 2 list the sequence data recovered from the 31 taxa.

**Figure 1.**
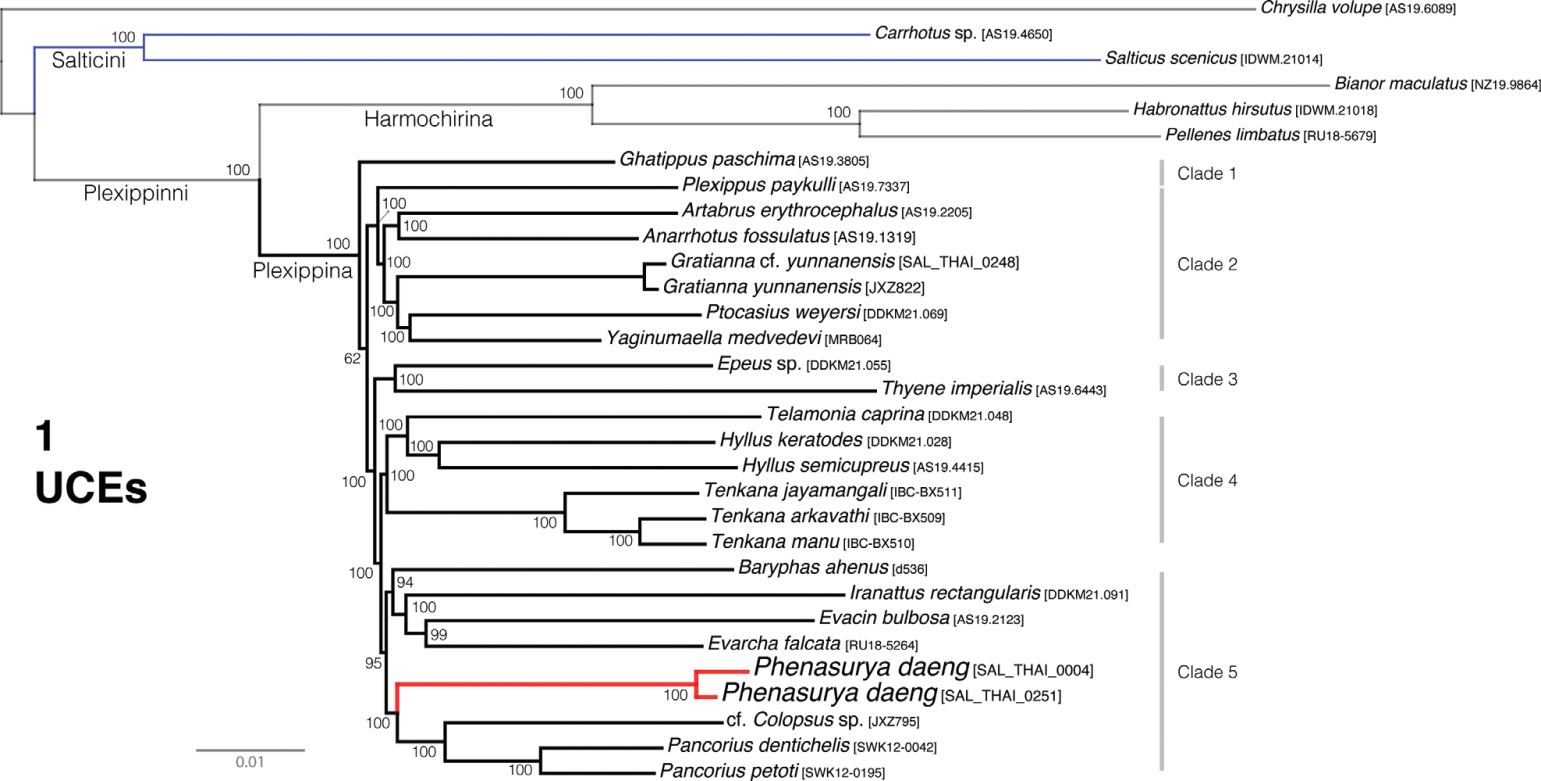
Maximum-likelihood tree from RAxML analysis (best of 10 replicates) of a concatenated dataset of 3031 UCE loci. Numbers at the nodes are the percentage recovery of the clade based on 1000 bootstrap replicates. *Phenasurya* (marked in red) is recovered as sister genus to cf. *Colopsus* and *Pancorius* of clade 5 and distantly from Salticini (marked in blue).

**Figure 2.**
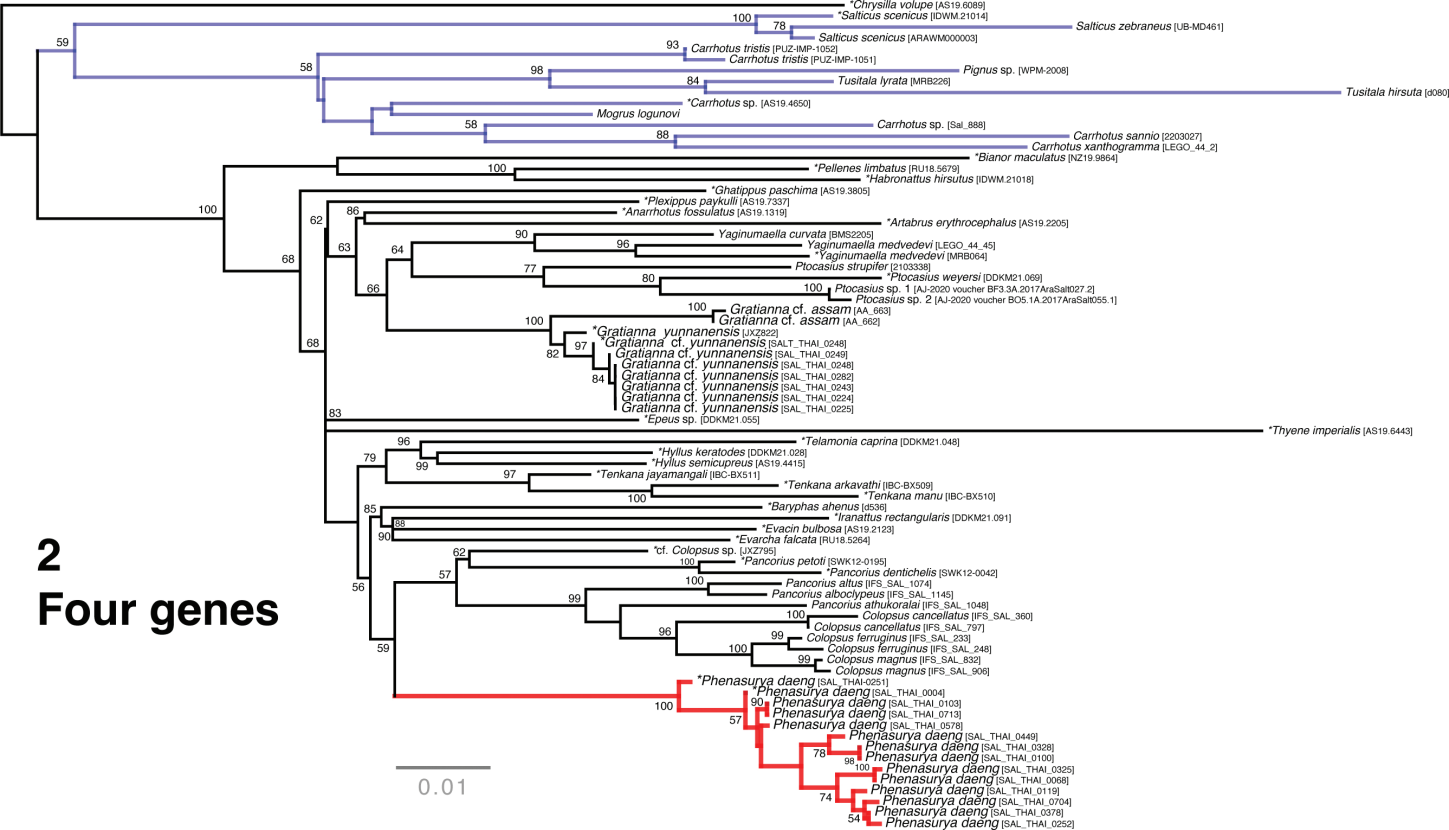
Maximum-likelihood tree from IQ-TREE analysis (best of 1000 replicates) of a concatenated, partitioned dataset of four genes (28S, 18S, H3, COI), constrained by the UCE topology. Node values indicate bootstrap support based on 1000 replicates. *Phenasurya* is recovered as monophyletic (bycatch + additional barcode data) and as sister to *Colopsus* and *Pancorius*. An asterisk preceding taxa denotes bycatch data corresponding to the UCE tree. *Phenasurya* (marked in red) is recovered well within Plexippina and are distantly placed from the expanded sampling of salticines (marked in blue).

The summary phylogenies from UCE and four-gene data are shown in Figs 1, 2. The broader relationships, the reciprocal monophyly of Plexippina and Harmochirina, as well as the generic relationships within Plexippina, are consistent with previous studies of comparable studies (Marathe et al. 2024a, 2024b, 2024c). As expected from high-volume data, most nodes exhibit robust bootstrap support. However, a notable deviation within the Plexippina clade is that *Ghatippus* Marathe & Maddison, 2024 is recovered as sister to the remaining plexippine taxa with strong support.

### ﻿DNA barcoding

The final COI alignment comprised 600 nucleotide positions (nps) from 21 specimens (Table 1). All nucleotide sequences were translatable into amino acid sequences without stop codons. Among the 600 nps, 92 were variable, with a transition-to-transversion ratio (R) of 1.73. The mean genetic distance (K2P) between *P. daeng* and G. cf. yunnanensis was 13.89% (SD = 1.56%). Intraspecific variation was low, averaging 0.05% (SD = 0.05%) for *G. yunnanensis* and higher 1.95% (SD = 0.38%) for *P. daeng*. The observed interspecific divergence is relatively high for jumping spiders (Trębicki et al. 2021) and falls within the range expected for clearly separated species.

### ﻿Taxonomic results

#### ﻿Family Salticidae Blackwall, 1841

**Subfamily Salticinae Blackwall, 1841**

**Tribe Plexippini Simon, 1901**

**Subtribe Plexippina Simon, 1901**

##### ﻿ Phenasurya

Taxon classificationAnimaliaAraneaeSalticidae

Marathe & Maddison gen. nov.

65923AE4-C63B-5311-8A51-BF2704B7683E

https://zoobank.org/3584E67C-5F2B-4CAE-A08B-3D08F31DFD5F

###### Type species.

*Phenasurya daeng* sp. nov., by monotypy.

###### Etymology.

The genus name *Phenasurya* is derived from the Greek “phen-”, referring to appearance, and the Thai “Surya”, referring to the sun. This alludes to the face of this elegant spider, red like the setting sun.

###### Remarks.

*Phenasurya daeng* is recovered as sister to cf. *Colopsus* (sensu Lin et al. 2024; Marathe et al. 2024c) and *Pancorius*: (*P. daeng*, (cf. *Colopsus*, (*P. dentichelis*, *P. petoti*))) (Figs 1, 2). Although *P. daeng* clusters near *Pancorius* and cf. *Colopsus*—both of which possess palps with a strongly sclerotised, thin embolus and an epigyne with distinctive ECPs—*P. daeng* exhibits markedly different genitalic morphology, including a male palp with an unsclerotised, jasmine-flower-bud-shaped embolus and an epigyne lacking distinctive ECPs. Consequently, *P. daeng* cannot be assigned to either *Colopsus* or *Pancorius* without synonymizing the two genera or, if included in one of them, rendering that genus paraphyletic. Therefore, the establishment of a new genus to accommodate *P. daeng* is justified, as it preserves the validity of both *Colopsus* and *Pancorius* and maintains the monophyly of each genus.

###### Diagnosis.

Unique among plexippines in possessing a relatively lightly sclerotised embolus resembling a jasmine flower bud—broad at the base and gradually tapering to a blunt tip. The embolus arises terminally from an ovoid, bulky tegulum with a lobe, extends retrolaterally, and terminates distally. In contrast, its close relatives cf. *Colopsus* and *Pancorius* typically have a highly sclerotised, longer, thinner embolus on a round tegulum.

The female epigyne also distinguishes *Phenasurya* from these genera: *Phenasurya* lacks distinctive ECPs, bearing instead a shallow medial notch at the epigastric furrow, whereas cf. *Colopsus* and *Pancorius* have distinctly divided ECPs positioned slightly anterior to the epigastric furrow.

The body markings of *Phenasurya* recall some Asian *Evarcha* (e.g., *E. bulbosa* Żabka, 1985) as well as smaller, red-faced African *Evarcha* (e.g., *E. culicivora* Wesołowska & Jackson, 2003). However, the red face of *Phenasurya* is absent in Asian *Evarcha*, and the epigyne clearly separates the genera: *Phenasurya* lacks ECPs, whereas *Evarcha* has distinctly divided ECPs. The male palp of *Phenasurya* also differs from that of African *Evarcha*, which typically has a well-sclerotised, much longer, thinner embolus and a bifurcated RTA.

The carapace markings may resemble those of *Burmattus*, but the male palp readily distinguishes the two. *Burmattus* has a highly sclerotised, curved cutlass-like embolus, a roundish tegulum lacking a distinctive lobe, a retrolateral cymbial groove, and a long, dorsally curved RTA. In addition, unlike *Phenasurya*, *Burmattus* bears a distinctive ECP positioned anteriorly and away from the epigastric furrow.

The body markings and male embolus of *Phenasurya* also resemble those of the unrelated salticine genus *Carrhotus*, particularly *C. qingzhaoae* and *C. taprobanicus*. However, in *Phenasurya* the embolus emerges prolaterally, leaning retrolaterally from a well-defined tegulum, whereas in *Carrhotus* the embolus is placed medially, on a somewhat irregular tegulum, giving the appearance of a candle flame (embolus) atop a candle (tegulum). The abdominal pattern also differs: *Phenasurya* has a simple medial band, while *Carrhotus* typically bears spotty abdominal markings.

###### Description.

Because this is a monotypic genus, the species description also applies to *Phenasurya*; see below.

##### ﻿ Phenasurya daeng

Taxon classificationAnimaliaAraneaeSalticidae

Marathe, Maddison & Trębicki sp. nov.

5D24E2E4-CB5C-5F43-95EB-12F4403E2A26

https://zoobank.org/2C849F30-EBC7-43BD-ACD2-4E72A082F4FE

Figs 3–8, 9–20

###### Type material.

Thailand • Kanchanaburi, Mueang Kanchanaburi District, Wang Dong; 14.145°N, 99.319°E; 987 m a.s.l.; 26 January–9 February 2020; coll. Ł. Trębicki. ***Holotype***: • ♂ (PSUZC-ARACH-022/SAL_THAI_0223), ***Paratypes***: • 1♀ (PSUZC-ARACH-013/SAL_THAI_0251) • 1♂ (PSUZC-ARACH-004/SAL_THAI_0004)

###### Additional paratypes.

2 ♂♂ & 4 ♀♀: Thailand • Kanchanaburi, Mueang Kanchanaburi District, Wang Dong; 14.145°N, 99.319°E; 987 m a.s.l.; 26 January–9 February 2020; coll. Ł. Trębicki.

###### Etymology.

The specific epithet *daeng* means “red” in Thai, alluding to the species’ distinctive red face.

###### Diagnosis.

Because this is a monotypic genus, the generic diagnosis also applies to *Phenasurya daeng*.

###### Description.

**Male** (holotype, PSUZC-ARACH-022). Measurements: Carapace 3.3 long, 2.5 wide. Abdomen 2.7 long, 1.7 wide. ***Carapace*** somewhat broad and relatively high, largely reddish-brown with the ocular area darker brown. Hints of white cheek bands from remaining scales originate at the junction of ALEs and AMEs, traverse just beneath the ocular ridge, and turn slightly upward at the slope of the ocular area. White marginal bands begin at the base of the ALEs and terminate at the posterior edge of the thoracic slope. ***Clypeus*** narrow, covered in orange-red hairs. ***Chelicerae*** narrow, brown, with one tooth having two tips (one conspicuous, one inconspicuous) on the retrolateral margin and two on the prolateral margin. ***Legs*** relatively long, robust, and dark brown. ***Palp*** as in Fig. 5, with an embolus flower-bud-shaped, broad basally and narrowing distally. Tegulum ovoid with a bulky tegular lobe. Abdomen relatively slender with a pale anteroposterior medial band extending only to midway on a dark, glossy integument.

**Figures 3–8.**
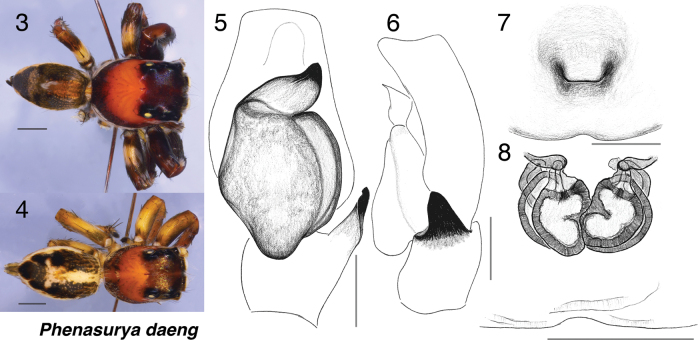
*Phenasurya daeng*. Bodies. 3. Male dorsal; 4. Female dorsal. Genitalia. 5. Male palp, ventral view; 6. Male palp, retrolateral view; 7. Female epigyne; 8. Female vulva. Scale bars: 1.0 mm (3, 4); 0.2 mm (5–8).

**Figures 9–20.**
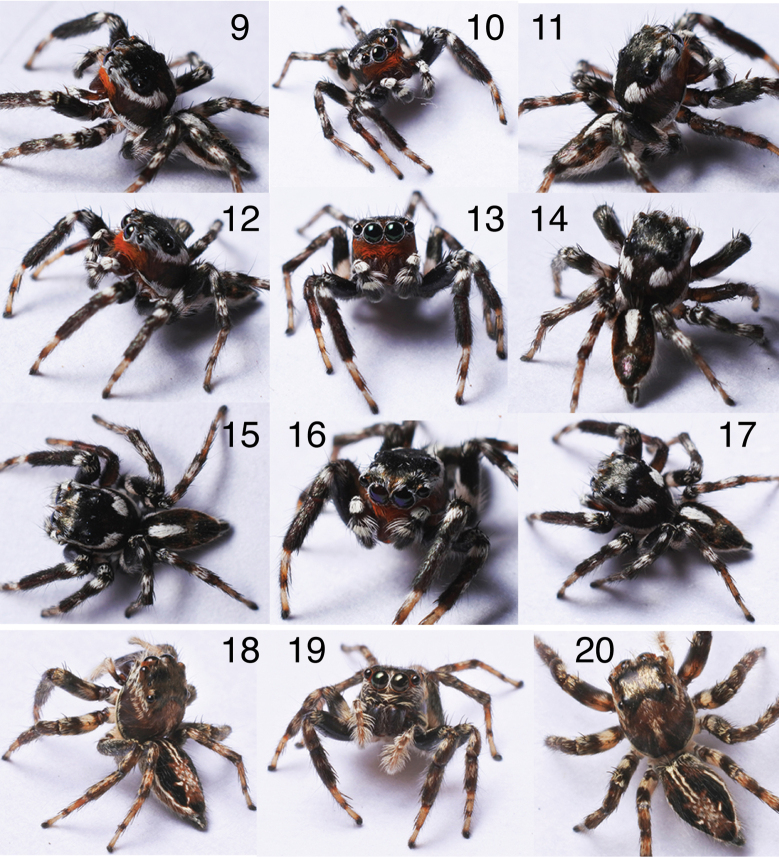
*Phenasurya daeng*, male (9–17) and female (18–20).

**Female** (paratype, PSUZC-ARACH-013). Measurements: Carapace 3 long, 2.2 wide; abdomen 3.3 long, 2 wide. ***Carapace*** similar in form and pattern to the male but overall paler. ***Clypeus*** narrow, sparsely covered with pale hairs. ***Chelicerae*** simple, vertical, brown. ***Legs*** relatively long, robust, and paler than in the male. ***Abdomen*** slender with a pale anteroposterior medial band extending to midway on a dark background, widening laterally near the posterior. Approximately three-quarters towards the posterior, a pair of dark, somewhat glossy patches occur on either side, with one larger patch at the posterior end. Spinnerets long. ***Epigyne*** as in Figs 7, 8, lacking distinctive pockets; epigastric furrow with a shallow medial notch.

###### Natural history.

**Figure 21.**
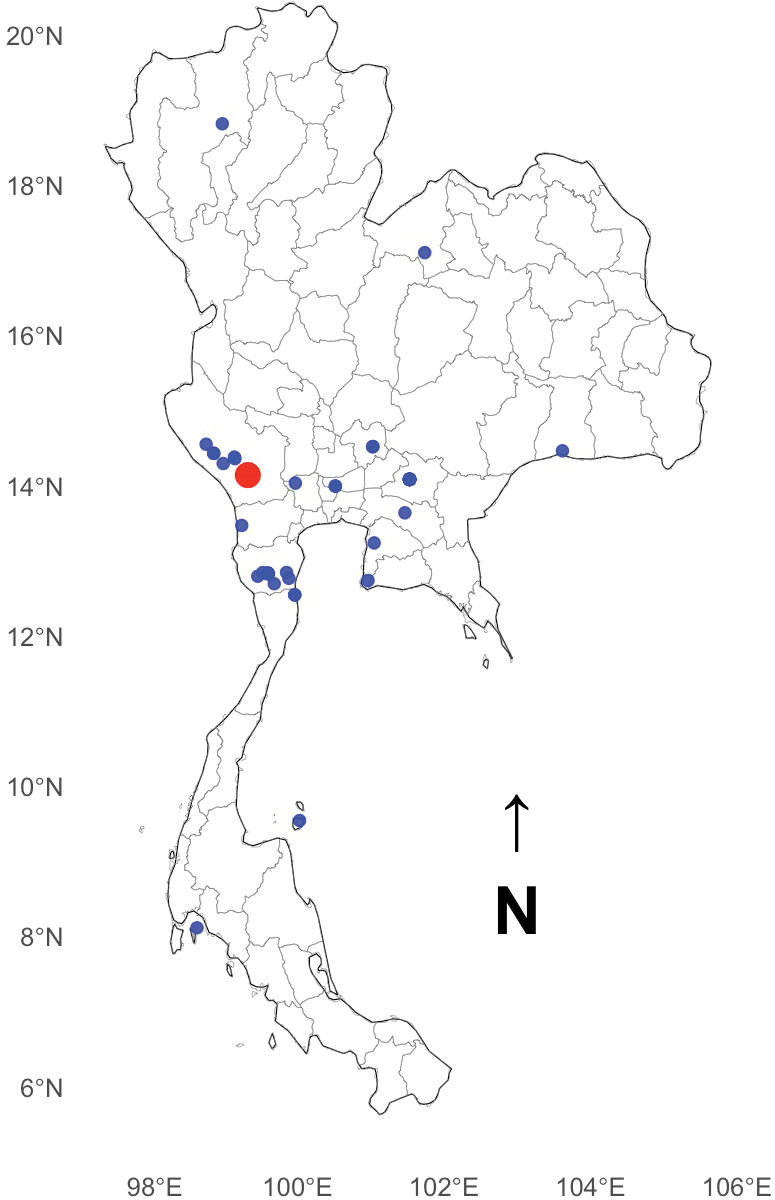
Distribution of *Phenasurya daeng*. Blue dots represent occurrence records based on iNaturalist data, and the bigger red dot indicates the type locality.

Widely distributed across Thailand (Fig. 21) and presently known only from that country, though its occurrence in neighbouring regions is likely given its range within Thailand. It is a vegetation dweller, typically found on low-lying to medium-high vegetation.

## Supplementary Material

XML Treatment for Phenasurya

XML Treatment for Phenasurya daeng

## ﻿Additional information

### Use of AI

AI was used only for language editing assistance. It had no role in the conception or development of the project and did not influence the data, results, or interpretations.

### Author ORCIDs

Kiran Marathe https://orcid.org/0000-0002-7364-3475

Łukasz Trębicki https://orcid.org/0000-0002-6384-226X

Katarzyna Janik-Superson https://orcid.org/0000-0001-9692-7238

Abdulloh Samoh https://orcid.org/0009-0005-8868-4607

Junxia Zhang https://orcid.org/0000-0003-2179-3954

Wayne P. Maddison https://orcid.org/0000-0003-4953-4575

### Data availability

All of the data that support the findings of this study are available in the main text or Supplementary Information.

## Supplementary materials

Supplementary material 1 Specifics of molecular data used for this phylogenomic analysis Data type xlsx Explanation note The file contains information regarding various details of UCE data of taxa used in the phylogenomic analysis. https://binary.pensoft.net/file/1463981This dataset is made available under the Open Database License (http://opendatacommons.org/licenses/odbl/1.0/). The Open Database License (ODbL) is a license agreement intended to allow users to freely share, modify, and use this Dataset while maintaining this same freedom for others, provided that the original source and author(s) are credited.Kiran Marathe, Wayne P. Maddison

Supplementary material 2 Accession numbers Data type xlsx Explanation note Accession numbers of nuclear 28S, 18S, H3, and mitochondrial COI fragments used in the four-gene phylogenetic analysis. https://binary.pensoft.net/file/1463982This dataset is made available under the Open Database License (http://opendatacommons.org/licenses/odbl/1.0/). The Open Database License (ODbL) is a license agreement intended to allow users to freely share, modify, and use this Dataset while maintaining this same freedom for others, provided that the original source and author(s) are credited.Kiran Marathe, Łukasz Trębicki, Wayne P. Maddison
